# Immunopathological mechanisms and targeted intervention strategies for chronic chikungunya arthritis: from viral persistence to autoimmunity

**DOI:** 10.3389/fimmu.2026.1775493

**Published:** 2026-04-27

**Authors:** Linsheng Zeng, Wenjie Lai, Yueyang Li, Meiling Yang, Jing Liang, Minhua Li, Zhizhun Mo, Yanjun Li, Yuxiang Liu, Xinzhi Li, Guangdong Tong

**Affiliations:** 1 Faculty of Chinese Medicine, Macau University of Science and Technology, Macao SAR, China; 2 Emergency Department, Shenzhen Traditional Chinese Medicine Hospital, Shenzhen, China; 3 School of Pharmacy, Faculty of Medicine and Laboratory of Drug Discovery from Natural Resources and Industrialization, Macau University of Science and Technology, Macao SAR, China; 4 The Fourth Clinical Medical College of Guangzhou University of Chinese Medicine, Shenzhen, China; 5 Department of Hepatology, Shenzhen Traditional Chinese Medicine Hospital, Shenzhen, China

**Keywords:** chronic chikungunya arthritis, immunometabolism, precision intervention, Treg/Th17 imbalance, viral persistence

## Abstract

Chronic chikungunya arthritis (CCA) is a major long-term sequela of Chikungunya virus (CHIKV) infection. Its pathogenesis involves persistent viral reservoirs in joint tissues, metabolic reprogramming of myeloid cells, dysregulated Treg/Th17 immunity, and autoimmune-like tissue injury. This review integrates recent advances in virology, immunometabolism, and clinical pathology to propose a four-stage pathological cascade: “viral persistence”, “myeloid metabolic dysregulation”, “Treg/Th17 imbalance”, and “autoimmunity-like injury”. Key cytokine networks and molecular interactions at each stage are summarized. Based on this model, we outline stage-specific therapeutic strategies, including antiviral agents targeting Mxra8 or nsP1 (Stage I), metabolic modulators such as HIF-1α inhibitors and IL-1β blockade (Stage II), immune-restorative therapies such as low-dose IL-2 and CTLA-4-Ig (Stage III), and anti-inflammatory biologics or DMARDs for RA-like pathology (Stage IV). This framework provides a mechanistic basis for precision intervention and supports the development of biomarkers and clinical trial designs for CCA.

## 1 Introduction

Chronic chikungunya arthritis (CCA) is the most prominent disabling sequela following Chikungunya virus (CHIKV) infection (1, 2). The acute phase of chikungunya infection is characterized by fever, rash, and severe polyarthralgia (3, 4); this is followed by persistent joint inflammation and pain that can last for months to years (5), while a subset of patients progress to CCA (6, 7). It is this subset of patients, who develop chronic arthritis following the typical acute articular symptoms, that is the primary focus of this review. While CHIKV infection can also lead to atypical manifestations (e.g., neurological or cardiac involvement) which influence prognosis, the immunopathological cascade driving the predominant sequela of CCA within the joints is the central theme of our discussion. This long-term functional impairment not only significantly reduces patients’ quality of life (7) but also poses an ongoing burden on global public health systems, as starkly evidenced by the recent 2025 outbreak in Guangdong, China (8). Despite this significant and evolving clinical challenge, the immunopathology of CCA remains incompletely understood. It is recognized to involve both viral persistence and immune dysregulation (9), yet the precise interplay and relative contributions of these factors, particularly the transition from persistent infection to autoimmune-like pathology, remain subjects of debate (10). Traditional research on CCA often focuses on isolated mechanisms, such as viral persistence or cytokine storms, failing to delineate the causal relationships and dynamic progression between them. To address this gap, this review systematically integrates recent advances from clinical pathology, molecular virology, and immunometabolism. We hereby propose a novel four-stage pathological cascade model that sequentially links “viral persistence,” “myeloid metabolic dysregulation,” “Treg/Th17 immune imbalance,” and “autoimmunity-like injury.” This integrative framework not only provides a coherent narrative for the complex immunopathology of CCA, but also establishes a rational basis for developing stage-specific therapeutic interventions, offering a fresh perspective for managing this debilitating condition and a potential research paradigm for other viral arthritides (11).

## 2 Viral biology and immune recognition of CHIKV: foundational aspects for chronicity

The Chikungunya virus (CHIKV) is a positive-sense, single-stranded RNA virus of the genus Alphavirus(family Togaviridae) whose genome encodes both the non-structural polyprotein (nsP1-4), primarily involved in replication and host immune modulation, and the structural polyprotein (Capsid-C, E3, E2, 6K, E1) which assembles to form the viral virion ( (12, 13).

### 2.1 Viral entry and receptor specificity

CHIKV infection is initiated by the binding of the viral envelope glycoprotein E2 to the host cellular receptor, Matrix Remodeling-Associated Protein 8 (Mxra8) (14). Mxra8 is highly expressed on joint tissue fibroblasts, osteoblasts, and chondrocytes, which largely explains the virus’s pronounced tropism for musculoskeletal tissues and the consequent arthritogenic phenotype (14, 15). Following receptor-mediated endocytosis, the low pH environment of the endosome triggers conformational changes in the E1 glycoprotein, leading to the fusion of the viral and endosomal membranes and the release of the viral nucleocapsid into the cytoplasm (16–18).

### 2.2 Viral replication and the roles of non-structural proteins

Upon uncoating, the viral genomic RNA serves as mRNA for the translation of the nsP polyprotein, which is processed into four individual nsPs (nsP1-4). These nsPs form the replication complex, responsible for the synthesis of negative-strand RNA intermediates and subsequent genomic and subgenomic RNA production (12). The subgenomic RNA directs the synthesis of the structural proteins. Beyond their essential roles in replication, specific nsPs are critical for evading host innate immunity, a key factor in establishing persistence. Notably, nsP1 possesses methyltransferase/guanylyltransferase activity, catalyzing the formation of a 5’m7GpppN cap on viral RNA, mimicking host mRNA to evade detection and enhance viral protein translation (19, 20). Furthermore, nsP2 has been shown to directly inhibit the presentation of Major Histocompatibility Complex class I (MHC-I) antigens, thereby impairing the recognition and clearance of infected cells by CD8+ T cells (21). These early immune evasion strategies are fundamental to the virus’s ability to establish a persistent reservoir.

### 2.3 Initial host immune sensing and the crossroad to chronicity

CHIKV is primarily sensed in the cytosol by RIG-I-like receptors, signaling via MAVS to induce type I IFN and ISGs, but additional pathways (cGAS–STING) also contribute (18, 22). The effectiveness of this initial response is a major determinant of viral control. However, as detailed in subsequent sections, CHIKV employs countermeasures (via nsP1/2) to dampen this response. Crucially, the detection of viral RNA (often via sensitive PCR assays in patient samples) must be distinguished from the presence of replication-competent virus, as the former may indicate residual viral material while the latter confirms an active, persistent infection that can continuously stimulate the immune system. It is the failure to completely eliminate replication-competent virus—particularly within immune-privileged niches like joint synovial macrophages and fibroblasts (23, 24) that shifts the host response from acute antiviral defense to the chronic immunopathology characterizing CCA. The sustained, low-level antigenic stimulation and the associated immunometabolic dysregulation form the basis of the pathological cascade described in this review.

### 2.4 Link to subsequent pathological stages

The foundational processes described above—specific tissue tropism via Mxra8, efficient replication, and early innate immune evasion—are prerequisites for the establishment of viral persistence (Stage I). The subsequent chronic inflammatory drive stems from the immune system’s ongoing attempt to respond to this persistent viral antigen, leading to myeloid metabolic dysregulation (Stage II), which ultimately disrupts adaptive immune homeostasis, causing Treg/Th17 imbalance (Stage III)​ and culminating in autoimmunity-like tissue injury (Stage IV).

## 3 Viral persistence: the core mechanism driving chronicity

**Figure 1:**
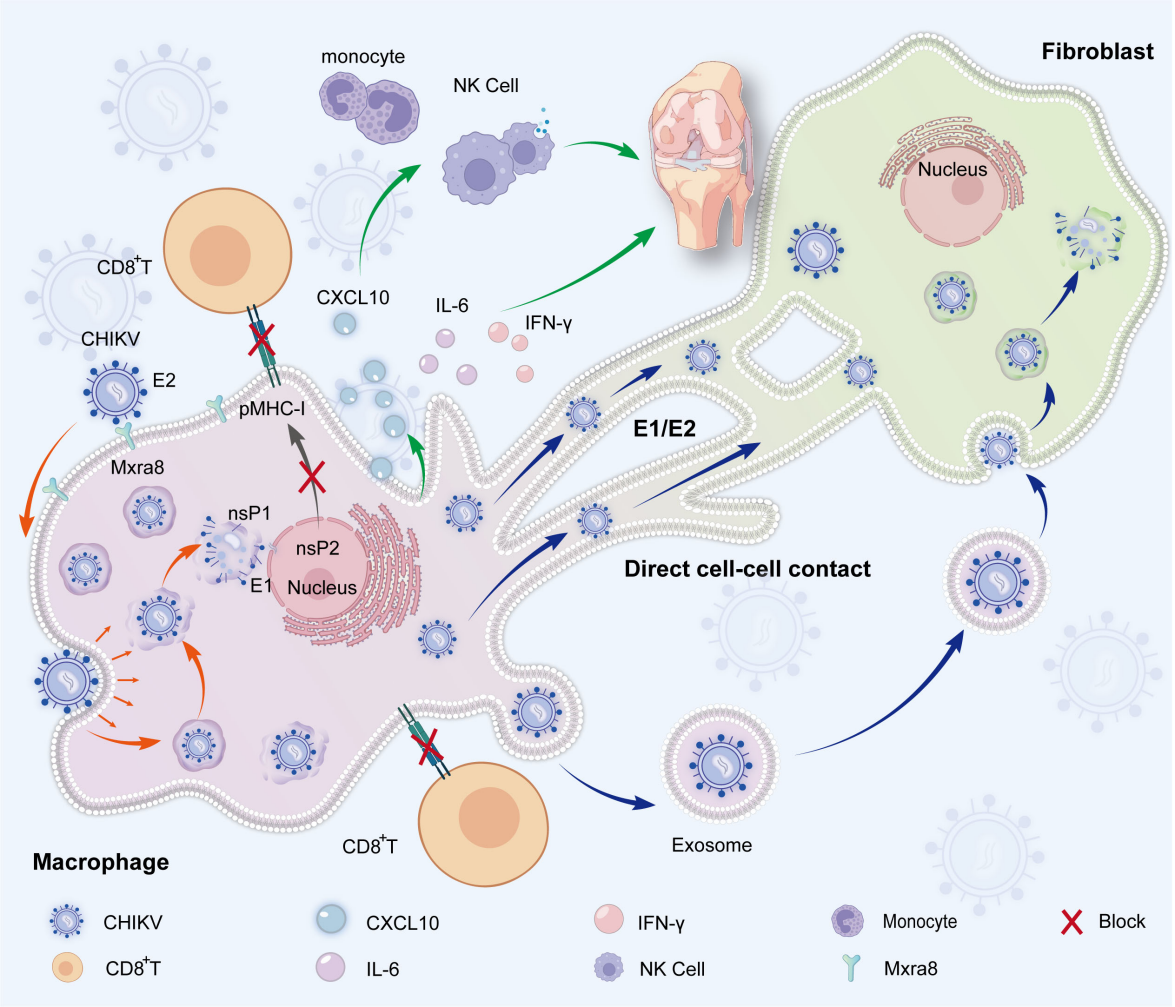
Mechanisms underlying persistent CHIKV infection. **(A)** CHIKV enters macrophages and replicates, thereby establishing a persistent viral reservoir (orange arrows). **(B)** The virus disseminates either directly through membrane nanotubes and cell–cell contact or indirectly via exosome-mediated transfer (blue arrows). **(C)** Infected macrophages secrete pro-inflammatory cytokines (e.g., IL-6, IFN-γ) and chemokines (e.g., CXCL10), which exacerbate synovial inflammation and recruit additional immune cells (green arrows). **(D)** Immune evasion strategies include nsP1-mediated mimicry of the host mRNA cap structure and nsP2-mediated suppression of MHC-I antigen presentation (black arrows).

The central pathological feature of CCA stems from immune evasion by CHIKV and persistent residence within joint tissue, driven by interconnected biological processes (**Figure 1**).

### 3.1 Cell-to-cell viral spread

CHIKV can spread directly between cells via cell-to-cell contact, a mechanism that evades humoral immune neutralization. Studies have shown that in cocultures of infected and uninfected cells, the virus can disseminate via this direct cellular route even in the presence of neutralizing antibodies, which is a process that is independent of the spread of free viral particles (25). Macrophages are a primary reservoir for long-term persistent CHIKV infection. In nonhuman primate models, the virus can be detected in joints, liver, and other tissues for months post-infection (26), where macrophages have been identified as key cellular reservoirs for persistence (23). In the synovial tissues of human patients with chronic CHIKV arthritis, the virus can persist long term in perivascular macrophages, serving as a “sanctuary” for viral replication (24). Fibroblasts are major target cells of CHIKV. In both human and animal models, the virus efficiently replicates in joint, muscle, and skin fibroblasts, leading to tissue damage and inflammation (27, 28). The colocalization of fibroblasts and macrophages in synovial tissue provides an anatomical basis for viral spread via membrane nanotubes or direct cell contact (22, 29). Membrane fusion proteins E1 and E2 play a pivotal role in the cell-to-cell spread of the virus. Specifically, the E2 protein facilitates attachment by binding to host receptors such as Mxra8. Subsequently, under the low-pH conditions within endosomes, the fusion peptide of E1 becomes exposed and actively drives the fusion between viral and cellular membranes (16–18). Cryo-electron microscopy studies have shown that CHIKV inserts E1 trimers into the target cell membrane to form fusion pores, enabling nucleocapsid release (30). Conformational changes in E2 domain A (e.g., the R82G mutation) can promote cell-to-cell spread, potentially by stabilizing intercellular protrusion contacts or modulating membrane fusion efficiency (31). Residues V178 and A226 in the E1 protein influence the pH threshold for membrane fusion and cholesterol dependence, optimizing the efficiency of viral spread between cells (17, 32). Beyond cellular protrusions, CHIKV-infected cells can release exosomes containing viral RNA and proteins, which can be taken up by neighboring cells, facilitating the transfer of viral components (33).

### 3.2 Receptor-driven tissue tropism

The envelope glycoprotein E2 of CHIKV specifically recognizes the host cell surface receptor Mxra8, a key mechanism underlying the virus’ tropism for joint tissues (14). Membrane fibroblasts within joint tissues are a major target cell; quantitative PCR (qPCR) detection has revealed that the Mxra8 expression level is approximately 15 times greater in these cells than in peripheral blood mononuclear cells (PBMCs). This difference in Mxra8 expression explains the viral targeting of joint tissues and the subsequent development of arthritis symptoms (14). Studies using cryo-electron microscopy and X-ray crystallography have shown that the Ig-like domains of Mxra8 bind with high affinity to domain B of the CHIKV E2 protein. Mxra8 adopts a unique disulfide-linked head-to-head conformation that wedges into a cleft formed by two adjacent E2-E1 heterodimers, creating a molecular interlocking structure (34). This binding mode is characteristic of arthritogenic alphaviruses. Mxra8 is expressed on fibroblasts, osteoblasts, chondrocytes, and skeletal muscle cells, supporting infection by CHIKV and other arthritogenic alphaviruses, such as Ross River virus and Mayaro virus (14, 15).

### 3.3 Viral reservoirs in immune-privileged microenvironments

CHIKV establishes viral reservoirs within the immune-privileged microenvironment of the joint synovium, primarily within macrophages and fibroblasts (22). Macrophages contribute to joint inflammation and tissue damage through the secretion of proinflammatory cytokines such as IL-6 and interferon-gamma (IFN-γ) and the release of chemokines (e.g., C-X-C motif chemokine ligand 10 [CXCL10]) that recruit immune cells such as monocytes and natural killer (NK) cells into synovial tissues (35). At the molecular level, the CHIKV nonstructural protein nsP1 catalyzes 5′m7GpppN RNA capping via the activity of its methyltransferases/guanylyltransferases, mimicking host mRNA to evade innate immunity and enhance viral protein translation (19, 20). CHIKV nonstructural protein 2 (nsP2), via its methyltransferase-like domain, directly inhibits the presentation of major histocompatibility complex class I (MHC-I) antigens, preventing viral peptides from being recognized by CD8^+^T cells (21). This immune evasion mechanism impairs the ability of CD8^+^T cells to clear infected cells. Furthermore, nsP2, a key component of the CHIKV replicase complex, is responsible for viral RNA synthesis and processing (e.g., nucleoside triphosphatase activity), thereby commandeering host cell resources to preferentially support viral replication (12).

## 4 Myeloid metabolic dysregulation amplifies inflammation

**Figure 2:**
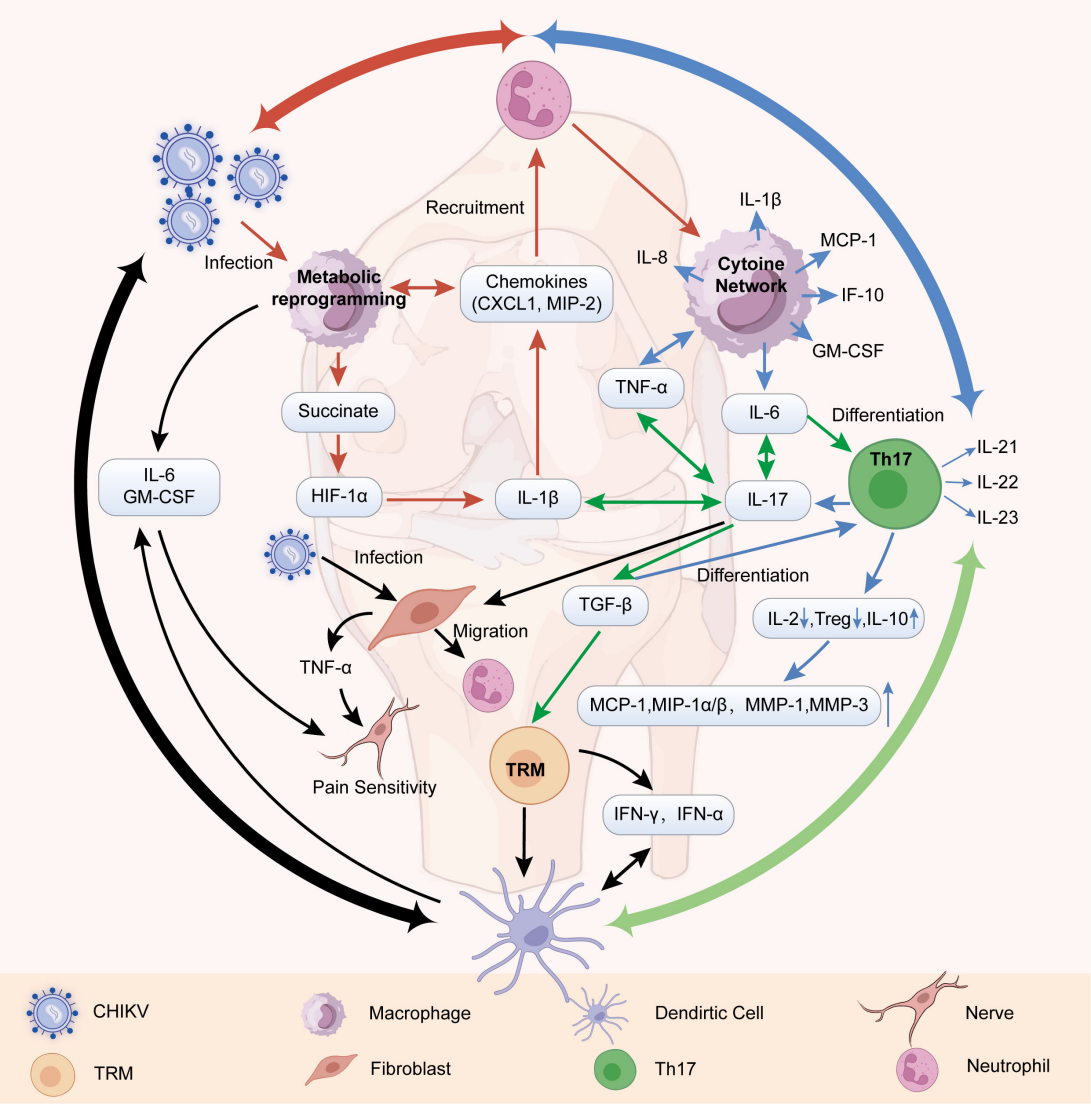
CHIKV infection of myeloid cells drives a persistent inflammatory circuit through metabolic dysregulation. **(A)** CHIKV infection of macrophages induces metabolic reprogramming, leading to succinate accumulation and stabilization of HIF-1α, which in turn activates IL-1β transcription and secretion, thereby initiating the core inflammatory cascade (orange arrows). **(B)** Myeloid metabolic abnormalities are tightly coupled with the cytokine network, characterized by marked elevation of pro-inflammatory mediators (e.g., IL-6, IL-8, TNF-α), hyperactivation of the Th17/IL-17 axis, and immune dysregulation manifested by reduced IL-2 production and diminished Treg frequency (blue arrows). **(C)** IL-17 signaling further amplifies the expression of IL-6, IL-1β, TNF-α, and TGF-β, with the synergistic effect of TGF-β and IL-6 driving the differentiation and expansion of TRM cells and Th17 cells (green arrows). **(D)** TRM cells secrete IFN-γ, TNF-α, and other cytokines, thereby activating dendritic cells and amplifying IL-6, GM-CSF, and IL-17–mediated inflammatory responses, ultimately establishing and perpetuating a pathological loop of chronic synovial inflammation and persistent arthralgia (black arrows).

The core mechanism underlying the chronicity of CCA lies in CHIKV infection-induced metabolic dysregulation in macrophages, which triggers the release of proinflammatory cytokines (e.g., IL-1β, IL-6, and IL-17). These cytokines (particularly IL-6 and Transforming Growth Factor-beta [TGF-β]) collectively shape the microenvironment and promote the differentiation of tissue-resident memory T (TRM) cells. Activated TRM cells secrete factors such as IFN-γ and TNF-α, forming a positive feedback inflammatory loop with macrophages and fibroblasts that collectively drives persistent joint inflammation and tissue destruction (**Figure 2**).

### 4.1 Myeloid metabolic dysregulation initiates the inflammatory core

#### 4.1.1 CHIKV infection triggers macrophage metabolic reprogramming to drive inflammation

A key pathological feature of CCA is the persistent inflammatory response that develops in the joint synovium, where macrophages act as crucial effector and regulatory cells. Significant infiltration of activated macrophages is often observed in the joint during both acute CHIKV infection and the subsequent chronic inflammation phase, driving the development of immunopathology (36). Evidence from proteomic and transcriptomic studies directly indicates that CHIKV infection causes profound, multidimensional, and temporally specific metabolic dysregulation in human macrophages (37, 38). Given the central role of the metabolic state in macrophage functional polarization and inflammation regulation (39), these observations suggest that CHIKV-induced metabolic alterations may play a significant role in the chronicity of CCA. Specifically, although the precise details of the metabolic changes in the joint microenvironment after CHIKV infection require further investigation, the metabolic dysregulation can be inferred to involve disturbances in key pathways on the basis of the general metabolic behavior of macrophages during inflammation. For instance, studies in other inflammatory models have revealed mechanisms through which metabolites (e.g., succinate) stabilize the transcription factor HIF-1α, activating the expression of downstream inflammatory genes (e.g., IL-1β) (40–42). Released IL-1β, a key proinflammatory mediator, further induces the production of other proinflammatory cytokines, such as chemokine (C-X-C motif) ligand 1 (CXCL1) and macrophage inflammatory protein-2 (MIP-2). These factors work together to recruit more macrophages and neutrophils to the joint, forming a positive feedback loop that amplifies the inflammatory response (43). These mechanistic insights suggest that metabolic dysregulation in macrophages is a core driver of chronic inflammation in CHIKV infection.

#### 4.1.2 Coupling of myeloid metabolic dysregulation and cytokine networks

Alterations in the metabolic pathways of myeloid cells are highly synchronized with the activation of inflammatory signaling pathways (37). The levels of inflammatory factors, including various inflammatory cytokines (e.g., IL-6, IL-8, IL-10, TNF-α, granulocyte–macrophage colony-stimulating factor [GM-CSF], and monocyte chemoattractant protein-1 [MCP-1] and IL-1β), in patients with CCA are elevated and highly correlated with disease activity and pain severity (44–48). During the chronic disease phase, the Th17/IL-17 axis is activated, and the expression of IL-17 and related factors (IL-21, IL-22, and IL-23) is significantly upregulated, promoting neutrophil infiltration and tissue inflammation, similar to the pathology of autoimmune diseases such as rheumatoid arthritis (RA) (49). Immune regulation is imbalanced, as characterized by reduced IL-2 levels and Treg numbers, leading to diminished immunosuppressive capacity and difficulty in terminating the inflammatory response; although the level of IL-10 is elevated, it is insufficient to fully suppress inflammation (44, 50). The levels of related chemokines and matrix enzymes, such as MCP-1 and MIP-1α/β, and matrix metalloproteinases (MMPs), such as MMP-1 and MMP-3, are elevated, promoting inflammatory cell recruitment and joint tissue destruction (46, 47). In simpler terms, CHIKV infection forces macrophages into a highly inflammatory metabolic state, causing them to release large amounts of cytokines that continuously recruit and activate additional immune cells. This creates a self-sustaining inflammatory loop within the joint.

### 4.2 Dynamic shifts in cytokine production shape the joint microenvironment and promote TRM cell differentiation

In chronic corrosive arthritis (CCA), persistent viral infection drives the sustained overexpression of inflammatory factors. Within this milieu, IL-17 signaling has been demonstrated to promote the production of IL-6, IL-1β, and TNF-α by inflammatory cells and to upregulate the expression of TGF-β, establishing a foundational pro-inflammatory network (51). TGF-β is a well-established core driver for the differentiation of tissue-resident memory T (TRM) cells (52). It is thus plausible that within the articular microenvironment of CCA, TGF-β similarly contributes to the activation and maintenance of TRM cells. Furthermore, extensive immunological research indicates that the combination of IL-6 and TGF-β synergistically promotes the differentiation of T helper 17 (Th17) cells (53, 54), whereas TNF-α can amplify IL-17 production by inducing monocytes to secrete IL-6 and IL-1β (53). Building upon these mechanistic insights, we posit that in the pathological context of CCA, activated TRM cells may form a positive feedback loop with synovial fibroblasts and macrophages through their cytotoxic effects and secretion of proinflammatory cytokines, collectively driving tissue destruction and the progression of chronic arthritis.

### 4.3 TRM cell feedback amplifies the inflammatory circuit

In RA, CD8+ tissue-resident memory T (TRM) cells have been identified as key drivers of disease chronicity and local flare-ups. They persist long-term in the joint synovium and can be rapidly reactivated by local stimuli, triggering recurrent inflammation (55). Although the direct identification and characterization of TRM cells in chronic chikungunya arthritis (CCA) remain to be fully established, their potential involvement is a compelling hypothesis based on several lines of reasoning. First, from a biological perspective, TRM cells are fundamental for establishing long-term immune surveillance at sites of previous viral infection, where they can rapidly amplify immune responses upon re-encounter with antigens (56, 57). Given that CCA is a sequela of acute CHIKV infection affecting the joints, it is plausible that virus-specific T cells differentiate into TRM populations and reside within the synovium. Second, the chronic, relapsing-remitting synovitis in CCA clinically mirrors aspects of RA (58), where TRM are established perpetrators. Therefore, analogous pathomechanisms involving local immune memory may be operative. If present in the synovium, TRM cells could sustain and amplify inflammation by secreting effector cytokines such as IFN-γ and TNF-α (56, 57), thereby contributing to the elevated levels of pro-inflammatory mediators like IL-6 and GM-CSF, which are strongly associated with persistent arthralgia in CCA (58, 59). This perpetuated inflammatory milieu can activate resident macrophages and fibroblasts, promoting a vicious cycle of tissue damage and pain sensitization. To move beyond speculation, future studies are most urgently needed to directly identify TRM cells in synovial tissue from CCA patients or animal models, and to functionally assess their role in sustaining chronic inflammation.

## 5 Autoimmune mechanisms

CCA is a complex immunologically mediated disease featuring viral persistence and notable autoimmune-like characteristics. Recent studies elucidate virus–host immune interactions underlying these features through refutation of classical autoimmunity, proposing molecular mimicry mechanisms and emphasizing the role of the Treg/Th17 imbalance in driving pathology.

### 5.1 Refuting the dominance of classical autoimmunity: lack of correlation between autoantibodies levels and disease severity

In traditional autoimmune diseases such as RA, autoantibodies (such as rheumatoid factor [RF] and anti-cyclic citrullinated peptide [CCP] antibodies) are often key markers of disease activity and severity (60–63). However, clinical studies in CCA patients have shown that the positivity rates for RF and CCP in CCA patient serum are significantly lower than those in RA patient serum and that their levels are not clearly correlated with disease severity or clinical manifestations (64, 65). Furthermore, CCA patients generally lack a characteristic autoantibody profile, suggesting that classical autoimmune responses are not the primary driver of chronic inflammation in CCA (66). These findings challenge traditional views, indicating that the autoimmune features of CCA may lean more toward abnormal immune activation induced by persistent virus rather than typical autoantibody-mediated autoimmunity.

### 5.2 Virus-mediated autoimmunity: from molecular mimicry to chronic inflammation

Recent research on molecular mimicry and immunology has revealed a new mechanism for triggering autoimmunity: cross-reactivity due to structural similarities between viral antigens and host self-proteins. The epitopes of the major envelope protein E2 of CHIKV and the host joint cartilage oligomeric matrix protein (COMP) are highly homologous (67). This structural similarity means that antibodies targeting the viral E2 protein, generated during infection, may mistakenly also attack the body’s own COMP protein-a phenomenon known as molecular mimicry. This cross-reaction can trigger a cascade of immune-mediated damage resembling autoimmune disease (26). Such cross-reactivity not only exacerbates local immune inflammation in the joints but also promotes the destruction of cartilage tissue, becoming a significant mechanism in the chronic joint pathology of CCA. This mechanism helps elucidate the potential link between viral persistence and autoimmune responses, and targeting the immunology of cross-reactions may become an important direction for future research.

### 5.3 Treg/Th17 immune imbalance: a key driver of chronic inflammation and autoimmune pathology in CCA

Immunoregulatory cell balance, particularly that between Tregs and proinflammatory Th17 cells, is crucial for maintaining immune homeostasis and preventing autoimmunity (68, 69). Studies have shown that in CCA patients, Tregs are not only reduced in number but also functionally impaired, with a significantly diminished immunosuppressive capacity (70–72). Key molecules such as CTLA-4 and Helios are downregulated in Tregs (73, 74). A pivotal factor underlying this Treg impairment is the insufficiency of IL-2, a cytokine critical for Treg survival, proliferation, and functional stability (75–77). A chronic inflammatory microenvironment driven by persistent viral antigens may directly contribute to IL-2 insufficiency by inducing T-cell exhaustion or altering antigen-presenting cell signaling pathways (46, 78). Concurrently, Th17 cell numbers and activity are increased, resulting in the secretion of large amounts of proinflammatory cytokines (e.g., IL-17) that drive local joint inflammation (74). The reduced level of IL-2, coupled with the inflammatory cytokine environment (elevated IL-6), further skews the immune balance towards Th17 dominance, as IL-2 is known to suppress Th17 differentiation (79, 80). Thus, Treg/Th17 imbalance not only sustains the chronic inflammatory state but also promotes the persistence of autoimmune-like pathological processes.

These findings underscore the therapeutic potential of restoring Treg function and modulating Th17 cell activity, providing new targets for immune intervention strategies in CCA. In other words, the loss of Treg-mediated immune control and the rise of Th17-driven inflammation shift the immune system toward a proinflammatory state that is difficult to reverse.

## 6 Integrated pathological model: the “viral persistence–myeloid metabolic dysregulation–Treg/Th17 imbalance-autoimmunity-like injury” cascade

**Figure 3:**
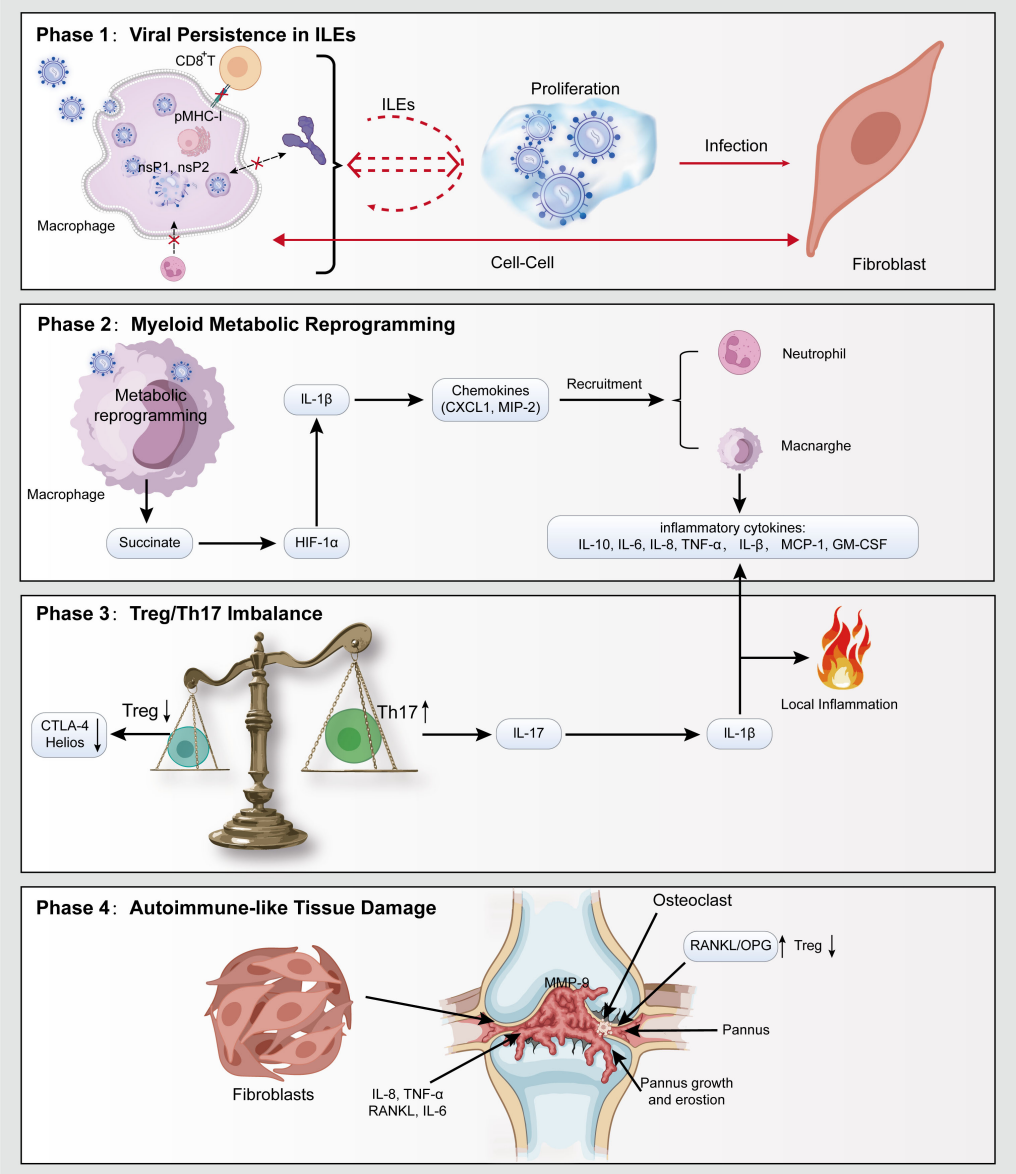
An integrated four-tier pathological model encompassing “viral persistence–myeloid metabolic dysregulation–Treg/Th17 immune imbalance–autoimmunity-like tissue damage.” Phase 1: CHIKV infects macrophages and evades host immunity through the actions of nonstructural proteins nsP1 and nsP2, while simultaneously spreading via direct cell-to-cell transmission between macrophages and fibroblasts, leading to the formation of ILEs. Phase 2: CHIKV infection induces metabolic dysregulation in macrophages, resulting in succinate accumulation and activation of proinflammatory cytokine release (e.g., CXCL1 and MIP-2). This process recruits additional macrophages and neutrophils, amplifying the local inflammatory cascade. Phase 3: In patients with CCA, both the number and function of Tregs are diminished, accompanied by downregulation of key molecules such as CTLA-4 and Helios, while Th17 cells expand and secrete abundant proinflammatory cytokines, jointly driving local joint inflammation. Phase 4: CHIKV infection activates synovial fibroblasts, leading to synovial hyperplasia and bone erosion. Elevated inflammatory cytokines further promote pannus formation and disrupt the RANKL/OPG balance, enhancing osteoclast activity and collectively contributing to joint destruction and chronic inflammation in CCA.

The complex immunopathology of CCA cannot be fully explained by a single mechanism. We propose an integrative four-stage model incorporating viral persistence in specialized tissue sites that harbor the virus (termed immune-licensed ecotopes, ILEs), myeloid metabolic dysregulation, Treg/Th17 immune imbalance, and autoimmune-like joint damage, clarifying their dynamic interrelationship (**Figure 3**). This staged model helps simplify a complex disease process by showing how each pathological step builds upon the previous one, ultimately leading to chronic joint injury.

### 6.1 Stage I: persistence of virus in ILEs

CHIKV maintains low-level viral persistence within joint-associated tissues, particularly residing in cell populations such as synovial macrophages and fibroblasts (81). These immune-sanctuary sites effectively protect the virus from clearance by the immune system by locally shielding it from immune surveillance, forming the basis for viral persistence (21).

### 6.2 Stage II: myeloid metabolic dysregulation drives inflammatory polarization

The persistent virus or its remnants within the joint can trigger profound changes in the metabolic state (reprogramming) of synovial immune cells, especially macrophages. Existing evidence suggests that such metabolic remodeling may involve the accumulation of key metabolites such as succinate and the activation of downstream signaling pathways, such as the stabilization and activation of HIF-1α, thereby promoting the production and release of key proinflammatory cytokines (e.g., IL-1β). The release of IL-1β not only exacerbates the intensity of the local inflammatory response but also, by inducing the expression of various downstream mediators, including chemokines (e.g., CXCL1 and MIP-2), forms a positive feedback loop that activates and recruits more myeloid cells to the joint, collectively sustaining and amplifying the chronic inflammatory state.

### 6.3 Stage III: Treg/Th17 immune imbalance

In CCA, immune homeostasis is disrupted, primarily manifested by a decrease in the number and functional impairment of immunosuppressive Tregs, while the number and activity of proinflammatory Th17 cells increase, resulting in the secretion of large amounts of inflammatory cytokines such as IL-17, driving local inflammation and autoimmunity-like pathology.

### 6.4 Stage IV: Autoimmunity-Like Injury

#### 6.4.1 Core mechanisms of RA-Like pathological features

a. Synovial hyperplasia and fibroblast activation

CHIKV infection can activate synovial fibroblasts, inducing the expression of MMPs such as MMP-9 and promoting synovial hyperplasia and subchondral bone erosion. Furthermore, infected synovial cells secrete chemokines such as Receptor Activator of Nuclear Factor Kappa-B Ligand (RANKL), IL-6, and IL-8, which attract and differentiate monocyte-macrophages into osteoclast-like cells, further exacerbating bone destruction (82, 83) (**Table 1**).

**Table 1:** Comparative table of core mechanisms and molecules in CCA and rheumatoid arthritis.

Mechanism/Feature	CCA presentation	RA presentation	Key molecules/cells	References
Synovial Hyperplasia	Marked	Marked	Fibroblasts, Matrix Metalloproteinase-9 (MMP-9)	(82, 83)
Pannus Formation	IL-6 driven, Angiogenesis	IL-6 driven, Angiogenesis	IL-6, Endothelial Cells	(44, 83)
Bone Destruction	RANKL↑, OPG↓, Osteoclasts↑	RANKL↑, OPG↓, Osteoclasts↑	RANKL, OPG, Osteoclasts	(82, 83)

b. Pannus formation and inflammatory cytokines

Inflammatory cytokines such as IL-6, whose levels are significantly elevated in CCA, promote endothelial cell migration and angiogenesis, leading to the formation of invasive pannus. Dynamic contrast-enhanced magnetic resonance imaging (DCE-MRI) studies have shown enhanced synovial vascular permeability, reflecting active inflammation (44, 83).

c. Bone destruction: RANKL/osteoprotegerin imbalance

CHIKV infection leads to upregulated RANKL expression and downregulated OPG expression, resulting in an increased RANKL/OPG ratio that promotes the differentiation and increases the activity of osteoclasts, causing bone erosion—a mechanism highly consistent with bone destruction in patients with RA (82, 83).

#### 6.4.2 Immune cells and cytokine network

Patients with CCA exhibit abnormal T-cell and natural killer (NK) cell function, persistently elevated levels of inflammatory cytokines such as TNF-α and IL-6, reduced numbers of Tregs, and impaired immune regulation, promoting chronic inflammation and tissue damage (44, 84).

## 7 Targeted therapy: mechanistic integration and clinical prospects of the four-stage pathological cascade

**Figure 4:**
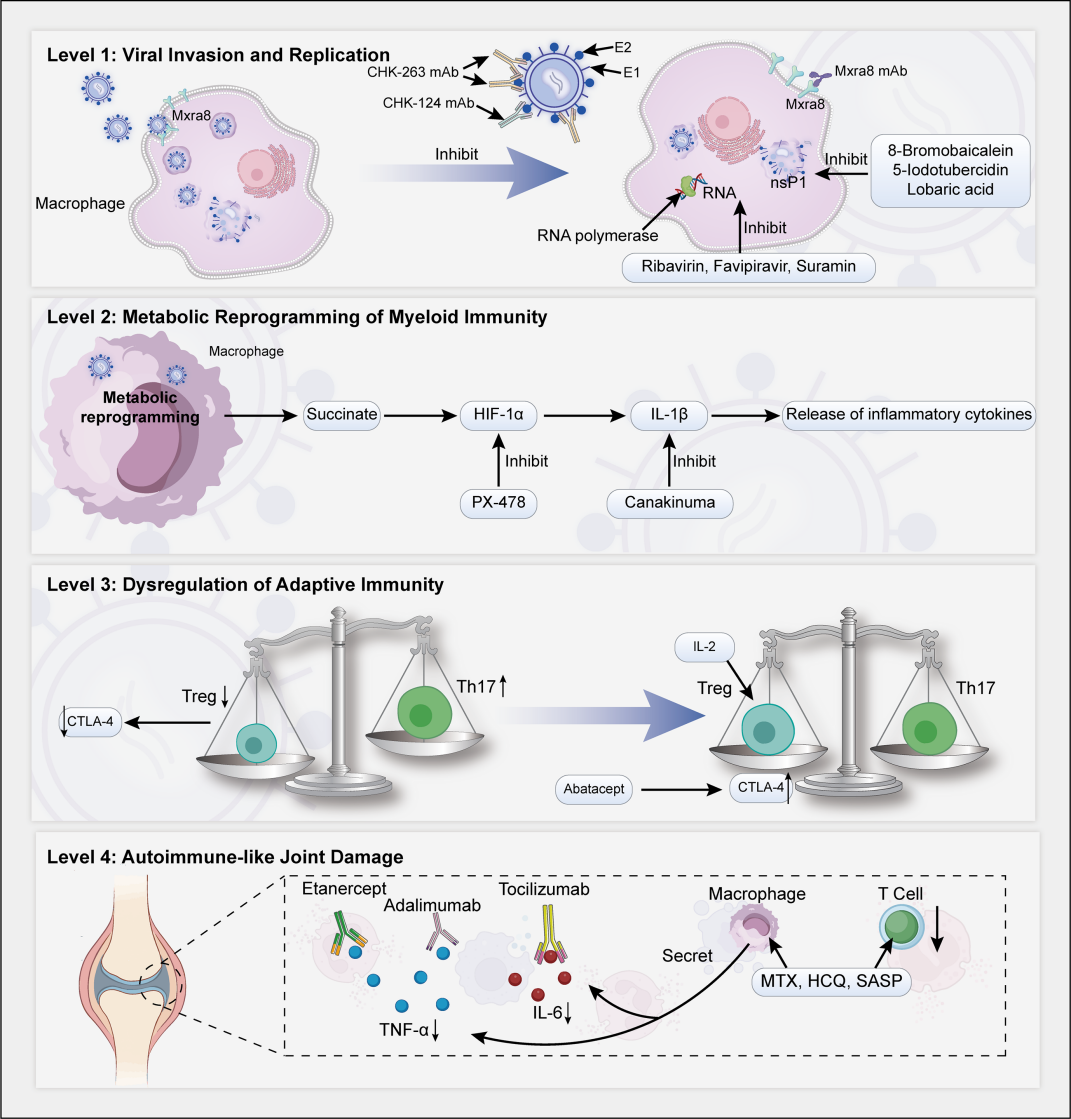
Therapeutic interventions targeting each stage of the four-tier pathological cascade. Level 1: Neutralizing antibodies targeting Mxra8/E1/2 (MXRA8 mAb, CHK-124, CHK-263) block viral entry, while nsP1 inhibitors (8-bromobaicalein, 5-iodotubercidin, lobaric acid) dismantle the immune-privileged replication complexes; in parallel, Ribavirin, Favipiravir, and Suramin interfere with RNA synthesis or viral assembly. Level 2: The HIF-1α–specific inhibitor PX-478 suppresses HIF-1αexpression, whereas the IL-1β monoclonal antibody Canakinumab neutralizes IL-1β; together, these agents converge to block downstream inflammatory signaling pathways. Level 3: Low-dose IL-2 selectively expands Tregs to restore immune tolerance, while CTLA-4-Ig (abatacept) concurrently restrains excessive effector Teff activation; synergistically, they re-establish the Treg/Teff balance. Level 4: TNF-αinhibitors (Etanercept, Adalimumab) block TNF-αactivity; IL-6 blockade (Tocilizumab) suppresses IL-6 signaling; and DMARDs, including MTX, HCQ, and SASP exert immunomodulatory effects by inhibiting immune cell activation and proinflammatory cytokine release.

As shown in **Figure 4**, the currently employed treatment for CCA primarily focuses on symptomatic relief, with the aim of alleviating pain and reducing joint inflammation using nonsteroidal anti-inflammatory drugs (NSAIDs) and short-term corticosteroids (85). However, given the chronic and persistent inflammatory nature of the disease, interest in the use of DMARDs and biologics that target specific inflammatory pathways is growing. On the basis of our proposed four-stage pathological model, multiple innovative treatment strategies have emerged, including antiviral, immunomodulatory, and receptor blockade approaches (**Table 2**). To make these mechanisms more clinically meaningful, each therapeutic strategy is aligned with the specific stage of the pathological cascade that it targets. This allows clinicians to better understand why certain treatments may be more effective during particular phases of the disease.

**Table 2:** Pathological mechanisms and corresponding treatment strategies for different stages of chikungunya virus infection.

Pathological stage	Therapeutic category	Specific target/drug	Mechanism of action	Experimental/clinical evidence	Prospects and challenges	References
I Viral persistence	Viral Receptor Blockade	Anti-Mxra8 mAb	Blocks CHIKV-E2 binding to Mxra8, inhibiting viral entry	Mouse: Pre-/post-exposure dosing → ↓Viral load & joint swelling; multi-cell model validation	Dose, timing, safety need optimization; nearing Phase I trials	(27, 29)
	Viral Receptor Blockade	Anti-E1/2 Neutralizing mAbs (CHK-124, CHK-263)	High-affinity E1/2 binding, blocking viral attachment/fusion/budding	Potent CHIKV neutralization *in vitro*/vivo, reduces viremia & arthritis	Complementary to Mxra8 mAbs; resistance monitoring needed	(83, 84)
	ILEs Inhibitors	nsP1 inhibitors: 8-bromobaicalein, 5‐iodotubercidin, lobaric acid	Inhibits nsP1 capping enzyme → disrupts ILEs, clears persistent virus	Mouse (oral 8-bromobaicalein): ↓Viral load & musculoskeletal inflammation; Lobaric acid inhibits GTP binding *in vitro*	First strategy targeting “immune privilege”; GLP tox & PK data needed	(90–92)
	Broad-Spectrum Antiviral	Ribavirin	Inhibits RNA synthesis	Effective in multiple cell lines, but high cytotoxicity; inconsistent animal/small clinical results	Narrow therapeutic window, limited clinical role	(93–96)
		Favipiravir	Prodrug inhibits RNA-dependent RNA polymerase (RdRp)	Effective in human cell lines, high oral bioavailability; resistant strains observed	Requires large Phase III trials & resistance monitoring	(93–95)
		Suramin	Multi-mechanism inhibition of CHIKV RNA synthesis	Effective against multiple strains *in vitro*, no cross-resistance 9494	Only *in vitro* data; *in vivo* PK/PD & safety unknown	(97)
II Myeloid cell metabolic dysregulation	HIF-1α Inhibition	PX-478	Downregulates HIF-1α transcriptional activity → inhibits myeloid inflammatory polarization	Effective in various inflammation models; no CHIKV-specific data	Needs validation in CHIKV models; potential synergy with metabolic modulators	(98)
	IL-1β Blockade	Canakinumab (anti–IL-1β mAb)	Neutralizes IL-1β → inhibits downstream inflammatory cascade	Approved for RA, significantly suppresses joint inflammation/bone destruction	No CCA studies; requires RCT design	(99)
III Treg/Th17 imbalance	Low-Dose IL-2	IL-2 (0.5–1 MIU/day)	Selectively expands Tregs, restores immune tolerance	Safe & effective in various autoimmune diseases; Tregs ↓ in CCA patients	Narrow dosing window; needs CCA-specific regimen & long-term follow-up	(100)
	CTLA-4-Ig	Abatacept	Blocks CD80/86 → upregulates Tregs/downregulates Teffs	Mouse arthritis model: ↓Joint swelling & T cell infiltration; synergizes with antiviral mAbs	Suitable for repurposing trials; infection risk monitoring needed	(101)
IV Autoimmunity-like injury	TNF-α Inhibition	Etanercept, Adalimumab	Neutralizes TNF-α → rapid anti-inflammatory & joint protection	Small CCA cohorts: pain & function improvement, but persistent symptoms in some	Large individual efficacy variation; needs high-quality RCT & TB reactivation monitoring	(105)
	IL-6 Blockade	Tocilizumab	Blocks IL-6 signaling → inhibits acute-phase response & bone destruction	Plasma IL-6 significantly ↑ in CCA; MTX (indirect IL-6 inhibition) effective	Lacks direct evidence; biomarker-driven trial design possible	(61, 107)
	Conventional DMARDs	Methotrexate (MTX)	Inhibits Dihydrofolate Reductase (DHFR) → ↓T cell proliferation, ↓IL-6/TNF-α	Retrospective/prospective studies: significant improvement in joint pain/swelling/function; combo with HCQ/SASP superior to monotherapy	Used off-label; needs RCT for optimal dose/duration & hepatorenal toxicity monitoring	(61, 108–115)
		Hydroxychloroquine (HCQ), Sulfasalazine (SASP)	Immunomodulation (specific mechanisms omitted)	Improved efficacy in combination with MTX	Serves as combination backbone; good safety, moderate evidence grade	(116)

### 7.1 Targeting viral receptors and immune-sanctuary sites: clearing persistent infection

#### 7.1.1 Mxra8-blocking antibodies

Anti-Mxra8 monoclonal antibodies demonstrate dual efficacy in a chikungunya arthritis animal model, exhibiting both potent antiviral and significant anti-inflammatory effects. Studies confirm that these antibodies inhibit viral cellular entry by blocking the interaction between CHIKV and its receptor, Mxra8. *In vivo*, they not only reduce viral load in joint tissues by 1–2 orders of magnitude but also attenuate joint swelling by 30-50% (23, 24). Preclinical studies on CHIKV-specific monoclonal antibodies further support that prophylactic administration markedly decreases systemic viral burden, suppresses viral dissemination, and significantly alleviates joint swelling (86, 87). These findings mechanistically suggest that such a strategy by blocking viral entry at the source can simultaneously inhibit viral replication and subsequent inflammatory pathology. The compelling preclinical evidence positions anti-Mxra8 monoclonal antibodies as a highly promising therapeutic candidate, warranting advancement into early-phase (Phase I/II) clinical trials, particularly for evaluation in patients during the “persistent viral phase” (Stage I), where viral RNA remains detectable in synovial fluid.

#### 7.1.2 Immune-licensed ecotope formation inhibitors

The CHIKV nsP1 protein promotes the formation of “immune-privileged sites” and viral replication complexes by remodeling the host cell cytoskeleton and membrane structures (83–86); the formation of these sites combined with existing immunosuppressive molecular mechanisms (22, 87) enable viral persistence and immune evasion. Inhibitors targeting the nsP1 protein significantly suppress CHIKV persistence in joint tissues. Various small-molecule inhibitors have been confirmed to effectively inhibit nsP1 activity, significantly reducing viral load in cell and animal models. Notably, the synthetic flavonoid derivative 8-bromobaicalein demonstrates potent antiviral activity *in vitro*, with a half-maximal effective concentration (EC50) of 0.49 µM against CHIKV replication. *In vivo* studies further confirm its therapeutic potential; oral administration (250 mg/kg, twice daily) in a murine model significantly reduces viral titers in footpad tissues and alleviates musculoskeletal inflammation, with plasma concentrations maintained above the effective threshold for over 12 hours without observable hepatorenal toxicity (88). Another promising candidate, the adenosine analog 5-iodotubercidin, functions by inhibiting the methyltransferase and guanylyltransferase activities of nsP1. It exhibits an *in vitro* EC50 of 0.409 µM and is capable of reducing intracellular CHIKV RNA levels by approximately 9-fold in infected cells (89). Furthermore, the natural lichen-derived metabolite lobaric acid competitively inhibits GTP binding to nsP1 with an inhibitory constant (Kᵢ) of 7.0 µM. It effectively curbs CHIKV replication in cell culture models, exhibiting EC50 values ranging from 5.3 to 8.9 µM and an average selectivity index (SI) of approximately 7 (90). Collectively, these compounds exert their antiviral effects primarily by impeding the crucial capping function of CHIKV nsP1, and they show significant promise in preclinical investigations. However, it is important to note that all three compounds remain in the preclinical development stage, and their progression into human clinical trials is pending further optimization and validation.

#### 7.1.3 Other antiviral drugs

In the screening for antiviral agents against CHIKV, numerous compounds have demonstrated potential in preclinical models. Notably, the efficacy of these agents exhibits a marked dependence on the cell line used for evaluation. For instance, ribavirin shows the most potent activity in HUH-7 human hepatoma cells (EC_50_=2.575 μg/mL), but its activity is significantly reduced in Vero and A549 cells (with EC_50_ values of 99.56 μg/mL and 117.1 μg/mL, respectively). Favipiravir also exhibits its highest potency in HUH-7 cells (EC_50_=20.00 μg/mL), whereas interferon-alpha (IFN-α) is most effective in A549 human alveolar epithelial cells (EC_50_ = 4.235 IU/mL) (91). Furthermore, a review article notes that other preclinical compounds, such as chloroquine (EC_50_=17.2 μM), arbidol (IC_50_<10 μg/mL), and 6-azauridine (EC_50_=0.82 μM), also possess antiviral activity against CHIKV (92).

Beyond monotherapy investigations, combination strategies have shown promise. Research indicates that the combination of favipiravir and interferon-alpha exerts synergistic antiviral effects in human-derived cell lines, including HT-1080 and SK-N-MC (93). On another front, the nonsteroidal anti-inflammatory drug mefenamic acid, when used alone, exhibits an EC_50_ of 13 μM against CHIKV. Remarkably, its combination with ribavirin (EC_50_=10 μM) at a 1:1 ratio significantly lowers the EC_50_ to 3 μM. This combination therapy also effectively reduces viral load in the blood (by 6.5-fold) and alleviates hepatosplenomegaly in a murine model (94).

The “drug repurposing” strategy is also noteworthy. For example, suramin, an approved anti-parasitic drug, effectively inhibits the replication of various CHIKV strains (EC_50_ ~75–80 μM) by targeting viral RNA synthesis (*in vitro* EC_50_ ~5 μM) and interfering with early post-attachment entry steps. It demonstrates a selectivity index (SI) greater than 60 and shows no cross-resistance to favipiravir- or ribavirin-resistant viral mutants, suggesting a distinct mechanism of action (95).

All of the aforementioned compounds remain in the preclinical research stage and have not yet advanced to human clinical trials. Collectively, these studies provide a critical data foundation and strategic direction for the subsequent optimization of candidate drugs, the design of combination strategies, and their ultimate translation into clinical applications.

### 7.2 Targeting myeloid metabolic dysregulation: blocking the succinate-HIF-1α inflammatory axis

#### 7.2.1 HIF-1α-specific inhibitors

HIF-1α is a core transcription factor that regulates cellular responses to hypoxia, inflammation, energy metabolism, and other pathophysiological processes. Its inhibitors (e.g., PX-478) can reduce HIF-1α expression or activity through various mechanisms, thereby suppressing inflammation and angiogenesis, and have shown anti-inflammatory potential in various inflammation models (96). Although the use of HIF-1α inhibitors for the treatment of CHIKV infection has not been evaluated directly, future applications could involve combination with metabolic modulators to precisely inhibit the polarization of proinflammatory myeloid cells.

#### 7.2.2 IL-1β blockade therapy

The IL-1β mAb canakinumab has shown significant clinical efficacy in the treatment of various inflammatory and autoimmune diseases. Its mechanism involves primarily neutralizing IL-1β and blocking downstream inflammatory signaling pathways, thereby reducing the release of proinflammatory cytokines and ameliorating tissue damage. For example, in RA, canakinumab effectively suppresses joint inflammation and bone destruction, significantly improving clinical symptoms and slowing the progression of joint damage (97). Although there are some similarities in inflammatory mechanisms between CCA and RA, no studies have directly evaluated the efficacy of IL-1β mAbs (e.g., canakinumab) in the treatment of chikungunya fever.

#### 7.2.3 Restoring regulatory T-cell function and immune tolerance

##### 7.2.3.1 Low-dose IL-2 therapy

Patients with chronic chikungunya arthritis (CCA) frequently demonstrate diminished regulatory T cell (Treg) numbers and compromised function, resulting in impaired immune tolerance. Studies have demonstrated that, in a murine model of post-CHIKV infection arthritis, treatment with an anti-IL-2 monoclonal antibody alone, by moderately extending the half-life of endogenous IL-2, appears more conducive to inducing a state of immune tolerance and thereby ameliorating joint pathology. In contrast, administration of high-dose IL-2, whether given alone or as an IL-2/anti-IL-2 antibody complex, concurrently expands Tregs and activates pathogenic effector T cells (Teff), ultimately attenuating the therapeutic benefit (98). All of these therapeutic approaches are currently in the preclinical research stage. This study provides preliminary experimental rationale for future exploration of anti-IL-2 monoclonal antibody as a potential immunomodulatory strategy for treating CHIKV-induced chronic arthritis.

##### 7.2.3.2 CTLA-4-Ig (abatacept treatment to reestablish the Treg/Teff balance)

Preclinical studies in animal models have demonstrated that initiating treatment at 3 days post-infection (coinciding with symptom onset) yields significant therapeutic effects. Monotherapy with CTLA4-Ig (Abatacept) significantly reduced foot swelling area from 9.0 mm² in the control group to 7.8 mm² (P tofacitinib decreased swelling from 9.3 mm² to 8.5 mm² (P (4N12) demonstrated synergistic efficacy. Compared to controls, the combination nearly ablated foot swelling (reducing it from 10.8 mm² to 6.6 mm², *P<0.0001*) and reduced infectious viral titers in the infected ankle by approximately 10,000-fold within two days of treatment (*P<0.0005*) (99).

This combined regimen also significantly lowered the levels of various pro-inflammatory cytokines/chemokines (e.g., CXCL10, MCP-1) in joint tissue and markedly reduced the infiltration of CD45+ leukocytes, CD4^+^ T cells, and inflammatory monocytes into the joints (99). It is important to note that this work constitutes a preclinical study, with all data derived from mouse models. While its findings—particularly the CTLA4-Ig/anti-viral antibody combination strategy—provide robust proof of concept for treating CHIKV arthritis, the primary contribution of this research lies in identifying candidate therapeutic approaches and elucidating their mechanisms of action, thereby laying a foundation for potential future human clinical trials.

### 7.3 Targeted strategies for autoimmunity-like injury

#### 7.3.1 TNF-α inhibitors

In the therapeutic strategy for CCA, immunomodulatory therapies targeting tumor necrosis factor-alpha (TNF-α) have demonstrated clear clinical value. Several TNF-α inhibitors, including infliximab, etanercept, adalimumab, certolizumab pegol, and golimumab, have received FDA approval for the treatment of autoimmune diseases (100). The application of these agents in CCA is primarily grounded in their potent anti-inflammatory mechanisms. Preclinical studies provide supportive evidence: the novel small-molecule compound benpyrine directly binds to and inhibits TNF-α activity (KD = 82.1 ± 5.0 μM; IC50 = 0.109 μM), effectively attenuating inflammation in arthritis models (101). Furthermore, treatment with etanercept in a collagen-induced arthritis (CIA) rat model not only reduced serum C-reactive protein (CRP) levels but also downregulated the expression of circulating microRNAs associated with endothelial dysfunction, namely miRNA-146a-5p and miRNA-155-5p, and improved vascular function. This suggests that TNF-α inhibitors may alleviate chronic joint symptoms by modulating vascular inflammation (102). Direct clinical evidence for CCA stems from a 13-year long-term follow-up study of patients post-CHIKV infection. The results showed that among patients with chronic chikungunya-associated inflammatory joint disease meeting the classification criteria for rheumatoid arthritis (RA), spondyloarthritis (SpA), or psoriatic arthritis (PsA), 56.7% (17/30) still exhibited persistent symptoms more than a decade after the initial infection. Within this long-term affected cohort, 17.6% (3/17) of patients continued to receive TNF inhibitors (such as adalimumab or infliximab) to manage their condition, while an additional 29.4% (5/17) were treated with methotrexate (99). This study delineates the indications for the use of TNF-α inhibitors in CCA: they are applicable to patients who develop post-infection arthritis with symptoms persisting for over three months and who clinically meet the classification criteria for specific inflammatory rheumatic diseases (99). Summarizing the drug development stages: Currently, TNF-α inhibitors used for CCA are all marketed biologics; however, their application constitutes off-label use, supported by evidence from observational clinical studies (99). In contrast, novel small-molecule inhibitors (benpyrine) and the modulation of specific miRNAs remain in the preclinical research stage and have not yet been evaluated in CCA patients (101, 102).

#### 7.3.2 IL-6 receptor blockade

Targeting the IL-6 signaling pathway represents a pathogenesis-based therapeutic strategy. Preclinical studies have confirmed that CHIKV replication within skeletal muscle cells drives local IL-6 release, constituting a key pathogenic event (103). Animal experiments demonstrated that infection with an engineered CHIKV strain with restricted skeletal muscle replication (SKE) significantly alleviates foot swelling and inflammation. Furthermore, IL-6 receptor blockade in mice infected with wild-type CHIKV reduces foot swelling to levels comparable to those observed in the SKE group (103). This indicates that IL-6 release triggered by viral replication is a central mediator of joint inflammation. However, applying IL-6 receptor blockers (tocilizumab) to CCA patients currently lacks support from prospective randomized controlled clinical trial data. Existing literature indicates that their use is considered off-label and is typically reserved as an empirical treatment option for patients who respond inadequately to first-line disease-modifying antirheumatic drugs (DMARDs), such as methotrexate (MTX), and whose clinical presentation aligns with specific inflammatory rheumatic disease patterns, such as rheumatoid arthritis (RA) or spondyloarthritis (SpA) (6, 93).

#### 7.3.3 Immunosuppressive effects and clinical applications of methotrexate and other conventional DMARDs

The management of CCA primarily relies on disease-modifying antirheumatic drugs (DMARDs). Given the clinical and pathogenetic similarities between CCA and rheumatoid arthritis, DMARDs such as methotrexate (MTX) have been introduced into clinical practice for CCA (104). Preclinical research provides partial theoretical support, indicating that at therapeutic concentrations, MTX does not impair the antiviral immune response of human synovial fibroblasts, suggesting its potential safety in the context of possible persistent viral infection (105). Clinical evidence demonstrates the efficacy of MTX in CCA. A prospective study of 133 Brazilian patients showed that a 4-week regimen of MTX (20 mg/week) and/or leflunomide combined with dexamethasone significantly reduced disease activity, with the mean 28-joint Disease Activity Score using the erythrocyte sedimentation rate (DAS28-ESR) decreasing from 6.0 to 2.7 and the mean pain visual analog scale (VAS) score dropping from 81.8 to 13.3; this clinical improvement was maintained for 5 months post-treatment (106). Retrospective analyses corroborate these findings, reporting a positive therapeutic response to MTX in 75% of treated patients (107), while sulfasalazine (SSZ) with or without MTX showed good efficacy in 71.4% and 12.5% of cases, respectively (108). A review article further summarizes treatment strategies, noting that hydroxychloroquine (HCQ) combined with corticosteroids or other DMARDs has been used successfully for chronic rheumatic manifestations, and that MTX and SSZ (alone or in combination) are also effective for chronic CCA (109). However, the use of all these DMARDs constitutes off-label use, as no therapy is formally approved for CCA (104). Current evidence, primarily derived from retrospective case series (107, 110) and uncontrolled prospective studies (106), indicates that these drugs are typically considered for patients with chronic inflammatory arthritis whose symptoms persist for more than three months and whose clinical presentation resembles patterns of rheumatoid arthritis or spondyloarthritis (104, 107). The field lacks large-scale, prospective, double-blind, placebo-controlled Phase III clinical trials (104, 111). A 24-week randomized, controlled, open-label trial provides higher-level evidence for combination therapy, demonstrating that for chronic persistent CCA, triple therapy with MTX, SSZ, and HCQ is significantly superior to HCQ monotherapy. The combination therapy group achieved a lower mean DAS28-ESR (3.39 ± 0.87 vs. 4.74 ± 0.65) and Health Assessment Questionnaire (HAQ) score (1.14 ± 0.31 vs. 1.88 ± 0.47) at 24 weeks (112).

## 8 Clinical translation: imaging and prognostic biomarkers

Building on the four-stage pathological model, identifying stage-specific biomarkers is crucial for precision management. Herein, we highlight biomarkers supported by robust evidence for diagnosing CCA, assessing its activity, and predicting prognosis.

### 8.1 Imaging biomarkers for synovial activity and structural damage

Musculoskeletal ultrasound (MSK-US) has emerged as a key bedside tool for objectively quantifying synovitis, effusion, and tenosynovitis in CCA. Its findings, particularly synovial hypertrophy and power Doppler signal, show strong correlation with clinical measures such as tender joint count, pain severity, and patient-reported outcomes, often outperforming serum inflammatory markers (113). The presence of persistent synovitis on US in the chronic phase is a predictor for progression to an RA-like phenotype. Magnetic resonance imaging (MRI), especially dynamic contrast-enhanced (DCE) MRI, provides superior sensitivity for detecting early bone erosions, bone marrow edema, and synovial thickening, offering insights into the inflammatory burden and structural damage that may not be apparent on clinical examination or US (114, 115).

### 8.2 Inflammatory and immune biomarkers

Among circulating biomarkers, interleukin-6 (IL-6) and tumor necrosis factor-alpha (TNF-α) are most consistently implicated in the pathogenesis and persistence of chronic CCA. IL-6 levels remain persistently elevated from the acute to the chronic phase and show a robust correlation with clinical disease activity scores, joint swelling, and pain (46, 49). Similarly, TNF-α, a key osteoclastogenic and pro-inflammatory cytokine, is significantly upregulated in chronic CCA patients and has been identified as a potential disease marker (49). The pathogenic role of TNF-α is further supported by the proven efficacy of anti-TNF-α therapies in other inflammatory arthritides, providing a rationale for its exploration in CCA. Other inflammatory mediators, including IL-1β, IL-8, monocyte chemoattractant protein-1 (MCP-1), and matrix metalloproteinases (MMPs), as well as acute-phase reactants like C-reactive protein (CRP) and the erythrocyte sedimentation rate (ESR), are also frequently reported to be elevated in chronic CCA and correlate with symptom severity (46, 49, 116, 117–123). Extram articular immune dysregulation, indicated by markers like hyperferritinemia, has also been associated with persistent arthralgia (124).

### 8.3 Viral and humoral immune biomarkers

Biomarkers related to the viral trigger and the host antibody response hold prognostic and stratifying value. A robust neutralizing antibody or IgG response during the acute phase is associated with a significantly reduced risk of progressing to chronic arthritis, marking it as a favorable prognostic factor (125). Conversely, the detection of CHIKV RNA in synovial fluid, albeit not universally present, is the most direct evidence defining the subgroup of patients in the “viral persistence” stage (Stage I) (82). This finding could be crucial for selecting candidates for targeted antiviral therapies (1).

## 9 Discussion

Although viral RNA or antigen persistence has been proposed as a potential contributor to the chronic phase of chikungunya arthritis, current evidence indicates that chronicity cannot be attributed to viral persistence alone. Instead, CCA represents a multifactorial pathological process in which several mechanisms act in parallel or sequentially. These include CHIKV-induced metabolic reprogramming of macrophages that amplifies inflammatory cytokine production, dysregulation of the Treg/Th17 axis that impairs immune resolution, and autoimmune-like tissue injury driven by sustained activation of synovial fibroblasts and tissue-resident memory T cells. While the possibility of latent or intermittent viral replication triggering immune-mediated pathology cannot be excluded, the chronic inflammatory state characteristic of CCA is more accurately understood as the result of converging immunometabolic and immunoregulatory disturbances rather than a single etiological factor. Building upon this multifactorial perspective, the present review integrates these distinct pathological processes into a unified immunopathological cascade model consisting sequentially of “viral persistence”, “myeloid metabolic dysregulation”, “Treg/Th17 imbalance”, and “autoimmunity-like injury”. This model not only links discrete pathological events into a coherent dynamic process but also provides a conceptual framework for understanding CCA as an immune-metabolic disease triggered by viral infection with autoimmune-like features.

### 9.1 Four-level cascade model: integrating information from multiple perspectives and theoretical innovations

Traditional CCA research often focuses on a single mechanism, such as the persistent presence of viruses or inflammatory cytokine storms, and fails to reveal the causal relationships between and temporal dynamics of various pathological events. The novel four-level cascade model proposed in this study integrates evidence from multiple disciplines, such as virology, immunohistochemistry, and rheumatology, and depicts the complete pathological course of CCA from initiation and amplification to chronicity. The core innovation of this model lies in its integration of the temporal dynamics and causal relationships of CCA. It continuously positions the virus as a chronic “starter engine” rather than an accompanying phenomenon. This position is supported by a wealth of evidence: CHIKV cleverly evades humoral immunity through intercellular transmission (such as through membrane nanotubes) and joint tissue tropism mediated by the specific receptor Mxra8, establishing an “immune exemption niche” (ILE) in synovial macrophages and fibroblasts (27–29, 31, 35). More importantly, the immune escape mechanism mediated by the viral nonstructural proteins nsP1 (which mimics the host mRNA cap structure) and nsP2 (which inhibits MHC-I antigen presentation) provides the molecular basis for the long-term latency of the virus (16–18, 22). The establishment of this stage fundamentally explains why local inflammation in the joints persists even after acute infection and provides a convincing theoretical basis for antiviral treatment in the chronic phase. The significance of this model is clear; it is crucial to develop sensitive molecular diagnostic methods for synovial fluid (RT-ddPCR detection of viral RNA and antigen capture detection) to identify patient subgroups in the “viral persistence” stage of the disease for treatment with targeted antiviral drugs (Mxra8-blocking monoclonal antibodies and nsP1 inhibitors).

The second (abnormal metabolism of myeloid cells) and third levels (Treg/Th17 imbalance) in the model are the “amplifiers” and “bridges”, respectively, that connect viral persistence with terminal organ damage. We emphasize that metabolic reprogramming of myeloid cells is not an independent pathological event but rather triggered by sustained stimulation from residual viral antigens or low-level replication. Among them, the activation of the succinic acid–HIF-1α–IL-1β axis is key. The accumulation of succinic acid not only directly promotes the activation of inflammasomes and the maturation of IL-1β but also amplifies glycolysis and the transcription of inflammatory genes by stabilizing HIF-1α, thereby forming a self-sustaining positive feedback inflammatory cycle (42–44). This mechanism links the simple antiviral immune response with the underlying cellular metabolic program, revealing the integral driving force of chronic inflammation in CCA. This persistent inflammatory microenvironment (high levels of IL-6, IL-1β, and TGF-β) directly disrupted the balance of adaptive immune cells. It leads to a decrease in the number and functional exhaustion of Treg cells (as evidenced by the downregulation of CTLA-4 and Helios) while promoting the expansion and activation of Th17 cells (71–74, 79). An imbalance in the Treg/Th17 ratio is a sign of disrupted immune tolerance, causing the immune system to redirect itself from clearing the virus to attacking its own joint tissue, thus transitioning to the fourth stage of “quasiautoimmune damage”.

### 9.2 In-depth mechanistic analysis: from molecular simulation to spectator activation

The characteristics of the fourth level, “autoimmune damage”, are highly similar to those of RA, including synovial hyperplasia, the formation of vascular opacities, and bone erosion mediated by a RANKL/OPG imbalance. However, our model emphasizes that the “autoimmune” nature of CCA is fundamentally different from that of typical RA. Clinical studies have shown that rheumatoid factor (RF) and anti-cyclic citrullinated peptide (CCP) antibody positivity rates are low in CCA patients and that their correlation with disease severity is weak (26, 66–67). These findings suggest that the classic humoral immunity mediated by autoantibodies is not the main mechanism involved. What is the mechanism driving this type of autoimmune damage? Our analysis suggests two main possibilities: molecular simulation (cross-reactivity) and inflammation-driven bystander activation. On the one hand, studies have shown that the CHIKV envelope protein E2 shares epitope homology with the host articular COMP (13). These findings indicate that antibodies against viral E2 may cross-react and attack joint cartilage, triggering a true autoimmune response (26). On the other hand, a more likely scenario is a strong and sustained innate immune response triggered by persistent viral and metabolic abnormalities, resulting in high concentrations of proinflammatory cytokines (such as TNF-α, IL-6, and IL-17) produced locally in the joints. This inflammatory environment can nonspecifically activate resident self-reactive T cells or B cells (bystander activation) or directly induce tissue damage through the induction of MMPs and osteoclast activation (48, 49, 66, 105). Although this type of damage appears to be similar to autoimmunity, its origin is “virus centered”. This distinction has important clinical significance: treatments targeting specific autoantibodies may have limited effectiveness in CCA, whereas strategies aimed at controlling viral persistence upstream and intrinsic immune inflammation may more effective.

### 9.3 Clinical translational significance: from subtyping diagnosis to precise intervention

The most direct value of the four-level model proposed in this review lies in guiding precise treatment on the basis of the disease stage. Our proposed strategy of “phase I antiviral therapy, phase II metabolic regulation, phase III immune reconstitution, and phase IV anti-inflammatory blockade” transforms complex pathological processes into actionable intervention nodes. For example, in the early stages or active recurrence of the disease, by detecting viral RNA or specific antigens in the joint fluid, patients with active virus can be identified, and targeted antiviral therapy using Mxra8-neutralizing antibodies or nsP1 inhibitors is expected to slow disease progression (23, 24, 82, 87–90, 109). For patients entering the chronic inflammation stage, if biomarkers (such as IL-1β, IL-6, and succinate levels) indicate that the metabolic–inflammatory axis is activated, HIF-1α inhibitors (such as PX-478) or IL-1β antagonists (such as canakinumab) can be used to disrupt the positive feedback loop (94, 112). When immune imbalance becomes the main contradiction (as evidenced by a significant decrease in Treg number and an increase in IL-17 levels), immunomodulatory therapies such as low-dose IL-2 or CTLA-4-Ig (abaiput) demonstrate unique advantages aimed at restoring immune tolerance rather than simply suppressing immunity (95, 113). For patients who already have severe RA-like lesions, successful treatment of RA can be used as a reference to control inflammation and joint destruction with biological agents such as methotrexate (MTX), anti TNF-α antibodies, or anti-IL-6R antibodies (6, 99, 101–104, 114–118). The key to implementing this precise strategy lies in the integrated application of biomarkers and imaging. Simple clinical symptom assessment is not sufficient for accurate staging. Musculoskeletal ultrasound (MSK-US) and dynamic contrast-enhanced magnetic resonance imaging (DCE-MRI) can be used to objectively quantify synovial inflammation, hyperplasia, and bone erosion (119–120, 125). Together, the levels of IL-6 and IL-17 in the circulation, the Treg/Th17 ratio, and the abundance of potential metabolites that may be validated in the future (such as succinic acid) form the basis for molecular stratification. By combining imaging, immune, viral, and metabolite biomarkers, a multidimensional disease activity scoring system can be constructed, thereby achieving truly personalized treatment.

### 9.4 Implications for prevention and prognostic assessment

The value of the cascade model established in this review goes far beyond guiding stratified treatment in the chronic phase. Its greater significance lies in providing an innovative theoretical framework and practical direction for disease prevention and prognosis assessment. Moving the intervention threshold from chronic phase management to the acute phase and even postexposure prevention is a fundamental strategy for reducing the overall disease burden of CCA. On the basis of the model, the key to successful chronic prevention lies in preventing the establishment of the first phase (viral persistence) or rapidly intervening at the second and third phases (immune metabolism imbalance and immune imbalance) after establishment to prevent the disease from progressing to the irreversible fourth phase (autoimmune damage). This finding suggests that during the acute infection period, the treatment plan should not be limited to symptomatic supportive treatment. For patients with high-risk factors for chronic disease, early combination treatment with antiviral and immunoregulatory agents should be considered. For example, the use of potent direct-acting antiviral drugs (such as anti-Mxra8 antibodies and nsp1 inhibitors) to rapidly clear the virus, combined with short-term, low-dose immunomodulators (such as low-dose IL-2, IL-1β receptor antagonists, or hydroxychloroquine), may be able to simultaneously inhibit virus replication and prevent immune tolerance damage caused by excessive inflammation (23, 24, 88–90, 94, 113, 121), thereby preventing the formation of an “immune exemption niche” and the initiation of subsequent cascade reactions. In terms of prognostic assessment, this model provides a clear logical basis for developing multidimensional predictive biomarker panels. Prognostic assessment should no longer rely on a single clinical symptom or common inflammatory markers (such as CRP levels). In contrast, multiple biomarkers, such as viral, immunometabolic, inflammatory, and immune cell function markers should be integrated to predict the risk of disease chronicity (33, 46, 49, 52, 66, 107, 126–130). Specifically, the sustained detection of viral RNA and antigens and the implementation of specific IgG avidity index testing in joint fluid or serum after the acute phase serves as direct evidence of the continued presence of the virus, indicating a high risk of chronicity. Moreover, sustained high levels of IL-1β, IL-6, succinic acid, or specific chemokines (such as MCP-1) in the serum during the acute or early recovery phase indicate metabolic disorders in myeloid cells and activation of inflammatory positive feedback loops, which are strong warning signals for disease chronicity (42, 45–50, 81, 105, 125–127, 130). In addition, early-stage exhaustion of Tregs (as evidenced by low expression of CTLA-4 and Helios) and the amplification of Th17 cells in peripheral blood serve as early indicators of immune imbalance, indicating an increased risk of the disease transitioning from the acute viral phase to the chronic immunopathological phase (71–74, 79).

By combining viral, metabolite, and immunological indicators with early imaging changes (such as persistent synovitis shown by ultrasound), a powerful prognostic prediction model can be constructed. This model can effectively distinguish between “self-limiting rehabilitation” and “chronic high-risk” patients in the early stages of the disease within 1–2 months after infection, thereby achieving early identification and precise reinforced intervention of high-risk populations, preventing irreversible joint injury and truly realizing the strategic shift from passive treatment to active prevention.

### 9.5 Current status and challenges in clinical trial translation

The therapeutic strategies summarized in **Table 2** are at various stages of development. Currently, most interventions remain in the preclinical or early clinical phase. For instance, Mxra8-neutralizing antibodies and nsP1 inhibitors (8-bromobaicalein) targeting Stage I have shown significant antiviral and anti-inflammatory efficacy in animal models, positioning them as prime candidates for future clinical trials (23, 24, 88). Therapies for Stages II and IV, such as the HIF-1α inhibitor (PX-478) or the IL-1β mAb (Canakinumab), while used in other inflammatory conditions, lack direct evidence in CCA and require targeted clinical validation (94, 112). Notably, some approved immunomodulators offer a pathway for rapid translation. For example, low-dose IL-2 and CTLA-4-Ig (Abatacept) for Stage III, and TNF-α inhibitors and IL-6 blockers (Tocilizumab) for Stage IV, have extensive safety and efficacy profiles in diseases like RA, making their “drug repurposing” for CCA an efficient strategy (95, 113). However, key challenges for precise translation remain: First, high-quality RCTs are urgently needed to confirm the efficacy of these strategies in CCA populations, ideally using adaptive designs aligned with different disease stages. Second, patient stratification using biomarkers (e.g., viral RNA, IL-6, Treg/Th17 ratio) is crucial to ensure the right therapy is delivered to the right patient subgroup at the right time. Finally, long-term safety, particularly when combining antiviral and immunomodulatory agents, requires careful monitoring. Future clinical research must focus on translating these promising strategies into standard care to improve outcomes for CCA patients.

### 9.6 Clinical phenotype divergence: chronic inflammatory arthropathy versus isolated neuropathic pain

One unresolved pivotal question in CHIKV infection is the differentiation of its chronic sequelae into distinct clinical phenotypes. Epidemiological studies reveal that approximately 25–60% of chronic patients develop a rheumatoid arthritis-like chronic inflammatory rheumatism (pCHIK-CIR), characterized by synovitis and structural joint damage (1, 2). Additionally, neuropathic pain symptoms (e.g., burning, electric shock-like sensations) are reported by 34.1% of patients. Among these, about 6.6% are classified as having a localized chronic musculoskeletal disorder predominantly featuring neuropathic pain (pCHIK-MSD), while 2.4% exhibit a clinical presentation in which neuropathic pain persists following the resolution of arthritic symptoms (3). The immunopathological cascade discussed in this review primarily elucidates the former, pCHIK-CIR. Progression to the inflammatory arthritis phenotype is associated with female sex, a high joint count during the acute phase (elevated RA score), prolonged viremia (>7 days), and persistent arthralgia/edema in the subacute phase (2, 4). It is driven by sustained high intra-articular levels of pro-inflammatory cytokines (IL-6, IL-8, TNF-α, IL-17), which exacerbate synovial immune infiltration, osteoclastogenesis (via RANKL/OPG imbalance), and MMPs-mediated tissue destruction (5–8). In contrast, the pathophysiological focus in the neuropathic pain-dominant phenotype (pCHIK-MSD) likely shifts toward sensitization within the peripheral and central nervous systems.

Low-grade systemic or perineural inflammation can sustain pain even in the absence of detectable synovitis or high local cytokine levels. Initial direct viral damage or intense inflammation may trigger small-fiber neuropathy (3). Subsequently, a peripheral nerve-immune interplay maintains neuropathic pain: injured nerves release mediators that recruit and activate macrophages, T cells, and mast cells. These cells, in turn, produce IL-6, TNF-α, IL-1β, chemokines, prostaglandins, and growth factors (9). These inflammatory mediators directly sensitize nociceptive neurons in the dorsal root ganglia and spinal cord, lowering their activation threshold, enhancing synaptic transmission, and activating glial cells. This process leads to central sensitization and a self-perpetuating cycle of “inflammatory neuropathic pain” (9, 10), explaining why neuropathic pain can persist in the absence of active joint inflammation. The effectiveness of gabapentinoids in approximately two-thirds of these patients further supports a neuropathic rather than a purely inflammatory mechanism (3).

The determinants of this phenotypic divergence may include differential viral tropism, host genetic and immunological predispositions, and the primary site of acute-phase injury. Future research correlating detailed cytokine profiles and neuro-immune biomarkers with clinical phenotypes is warranted to fully elucidate these distinct pathogenic trajectories.

### 9.7 Limitations and future prospects: translating insights from models to human disease

Although this model provides a clear framework, research on Chronic Chikungunya Arthritis (CCA) continues to face numerous challenges. A key limitation, as highlighted in the discussion on immunometabolism (Section 3.1.1), lies in the necessity to distinguish between mechanisms directly demonstrated in Chikungunya virus (CHIKV) infection and those extrapolated from related pathologies. While CHIKV infection has been confirmed to induce profound metabolic dysregulation in macrophages, and the critical “succinate-HIF-1α-IL-1β” inflammatory axis has been established in vitro using lipopolysaccharide (LPS)-activated macrophage models (43, 44), the pivotal role of this succinate-HIF-1α-IL-1β axis within the context of the human CHIKV-infected joint remains a strongly supported yet unvalidated hypothesis. Consequently, future studies must prioritize the direct validation of this and other inferred immunometabolic pathways in human synovial tissues to confirm their function as core drivers in CCA pathogenesis.

While the four-stage cascade model provides a coherent framework, CCA research still faces significant challenges, particularly in translating findings from animal and in vitro models to the complexity of human disease. Several key conflicts and knowledge gaps must be addressed to drive future research.

#### 9.7.1 Disparities between model systems and human disease

First, there are apparent disparities that underscore the need for cautious extrapolation. Key immunometabolic pathways identified as central in mice, such as the succinate-HIF-1α-IL-1β axis in myeloid cells, require direct validation in human synovial tissues. Whether the cellular sources, relative importance, and complete maps of these metabolic shifts are identical in patients remains an open question. More fundamentally, the specific mechanisms of viral latency, including how the virus may utilize host cell epigenetic regulation to establish silent infection, are still unclear. Similarly, the precise mechanisms driving Treg functional exhaustion and the regulatory networks through which Th17 cells influence long-term viral persistence in the joint need further elucidation.

#### 9.7.2 The critical challenge of disease heterogeneity

Furthermore, the striking clinical heterogeneity observed in CCA patients ranging from RA-like symmetric polyarthritis to fibromyalgia-like presentations suggests that the proposed cascade may not proceed uniformly in all individuals. It is plausible that distinct patient endotypes exist, characterized by the varying dominance of one pathological stage over others (a “viral-persistence-dominant” vs. an “autoimmunity-like-dominant” endotype). Current evidence, largely from aggregated patient groups, fails to explain this heterogeneity, representing a major gap between model-derived pathophysiology and the human clinical spectrum.

#### 9.7.3 Definitive knowledge gaps for future research

These translational challenges point to three definitive and interconnected knowledge gaps that future research must prioritize:

a. A deep mechanistic gap in human tissues: There is an urgent need for detailed spatial and single-cell multi-omics analyses of synovium and synovial fluid from well-characterized CCA patients. Such studies are essential to conclusively map the immunometabolic landscape, viral reservoir localization, and immune cell interactions directly within the human joint, thereby validating and refining the model.

b. A biomarker and endotyping gap: Correlating the molecular features from deep tissue analyses with detailed clinical and imaging phenotypes is crucial for developing biomarker-based stratification tools. Future research must use multi-omics techniques to conduct precise molecular subtyping of CCA patients. Identifying which patients are in which pathological “stage” or belong to which pathogenic endotype is the prerequisite for testing phenotype-specific and stage-specific interventions.

c. A targeted clinical trial gap: Perhaps the most significant translational gap is the lack of prospective, mechanism-informed clinical trials. Currently, most evidence for treatment strategies comes from mouse models or small-scale observations. It is urgent to design rigorous randomized controlled trials (RCTs), especially adaptive trials for different disease stages, to verify the efficacy and safety of targeted therapies. Future studies must evaluate whether stage-targeted therapies (antivirals for Stage I, immunomodulators for Stage III) outperform standard care in appropriately stratified patients. The efficacy and optimal timing of combining antiviral and immunomodulatory agents, a promising approach suggested from models, require rigorous clinical testing. An RCT evaluating such combination therapy versus standard care (NSAIDs) would be a milestone. Furthermore, beyond the chronic phase, the initial acute CHIKV infection itself presents with a spectrum of manifestations, ranging from the typical febrile polyarthralgia to severe atypical organ involvement. This clinical heterogeneity underscores the complexity of virus-host interactions. Indeed, disease heterogeneity needs to be taken seriously even within CCA. Patients present with different chronic clinical phenotypes, such as RA-like and fibromyalgia-like phenotypes. Future research should aim to link these diverse acute presentations and chronic outcomes to underlying variations in the immunopathological pathways discussed herein.

Finally, long-term prevention and control strategies cannot be ignored. Beyond treatment, and based on a deeper understanding of CCA pathogenesis, exploring early intervention measures to prevent chronicity (such as the use of immunomodulatory antiviral drugs in the acute phase) and developing early warning models to predict disease outcomes are crucial for reducing the overall disease burden of CCA.

Addressing these gaps by focusing on human tissue-based mechanistic studies, data-driven patient stratification strategies, and mechanism-informed trial design will be pivotal in moving the field from a compelling pathogenic model to effective, personalized therapies for CCA patients.

## 10 Conclusion

In summary, the four-tiered cascade model of chronic chikungunya arthritis (CCA) established in this review, which includes viral persistence, abnormal myeloid cell metabolism, Treg/Th17 imbalance, and autoimmune damage, provides an integrated and dynamic theoretical framework for a comprehensive understanding of the pathogenesis of this disease. This model profoundly reveals CCA as a virus-driven immune metabolic disease, emphasizing the necessity of multiphase intervention from viral clearance to immune reconstruction. By closely integrating basic research findings with clinical translation prospects, this article not only highlights the direction for developing staged targeted treatment strategies but also presents a paradigm for other studies on viral arthritis that can be referenced. Future research should focus on understanding the underlying mechanisms, promoting precise clinical trials, addressing disease heterogeneity, and ultimately translating scientific insights into effective solutions that can alleviate patient pain and reduce the social burden of this disease.

## Glossary

CCA: Chronic Chikungunya Arthritis
CHIKV: Chikungunya virus
Treg: Regulatory T Cell
Th17: T helper 17 cell
Mxra8: Matrix Remodeling-Associated Protein 8
nsP1/nsP2: Non-Structural Protein ½
IL: Interleukin (e.g., IL-1β, IL-2, IL-6,IL-8,IL-10, IL-17)
HIF-1α: Hypoxia-Inducible Factor-1-alpha
TNF-α: Tumor Necrosis Factor-alpha
TGF-β: Transforming Growth Factor-beta
IFN-γ: Interferon-gamma
TRM: Tissue-Resident Memory T cell
GM-CSF: Granulocyte-Macrophage Colony-Stimulating Factor
MCP-1: Monocyte Chemoattractant Protein-1
MIP: Macrophage Inflammatory Protein(e.g., MIP-2, MIP-1α/β)
MMP: Matrix Metalloproteinase(e.g., MMP-1, MMP-3, MMP-9)
CTLA-4-Ig: Cytotoxic T-Lymphocyte-Associated Protein 4-Immunoglobulin
DMARDs: Disease-Modifying Antirheumatic Drugs(e.g., MTX, HCQ, SASP)
MTX: Methotrexate
RA: Rheumatoid Arthritis
RF: Rheumatoid Factor
CCP: Cyclic Citrullinated Peptide
COMP: Cartilage Oligomeric Matrix Protein
RANKL: Receptor Activator of Nuclear Factor Kappa-B Ligand
OPG: Osteoprotegerin
MHC-I: Major Histocompatibility Complex Class I
pMHC-I: peptide–MHC class I complex
PBMCs: Peripheral Blood Mononuclear Cells
NSAIDs: Non-Steroidal Anti-Inflammatory Drugs
mAb: Monoclonal Antibody
GTP: Guanosine Triphosphate
MSK-US: Musculoskeletal Ultrasound
MRI: Magnetic Resonance Imaging
DCE-MRI: Dynamic Contrast-Enhanced Magnetic Resonance Imaging
CRP: C-Reactive Protein
ESR: Erythrocyte Sedimentation Rate
NK Cell: Natural Killer Cell
IgG/IgM: Immunoglobulin G/M
qPCR: Quantitative Polymerase Chain Reaction
RNA: Ribonucleic Acid
Teff: Effector T cell
CXCL: C-X-C Motif Chemokine Ligand(e.g., CXCL10, CXCL1)
E1: Envelope protein 1
E2: Envelope protein 2
ILEs: Immune-licensed ecotopes
cGAS: cyclic GMP-AMP synthase
SAM: S-adenosylmethionine
RRV: Ross River virus
RdRp: RNA-dependent RNA polymerase
mTOR: mechanistic target of rapamycin
HCQ: Hydroxychloroquine
SASP: Sulfasalazine
pCHIK-CIR: Post-Chikungunya Chronic Inflammatory Rheumatism
pCHIK-MSD: Post-Chikungunya Chronic Musculoskeletal Disorder
Therapeutic Agents/Compounds Mentioned: PX-478, Small-molecule HIF-1α
Canakinumab: Anti-IL-1βmonoclonal antibody
Abatacept: CTLA-4-Ig fusion protein (trade name)
CHK-124/CHK-263: Examples of anti-CHIKV E2 neutralizing monoclonal antibodies
Kᵢ: Inhibitory Constant
SI: Selectivity Index
HUH-7: Human Hepatoma-7
Vero: African Green Monkey Kidney Epithelial Cells
A549: Human Alveolar Epithelial Carcinoma Cells
HT-1080: Human Fibrosarcoma Cells
SK-N-MC: Human Neuroepithelioma Cells
4N12: a neutralizing human monoclonal antibody against CHIKV
JAK: Janus Kinase
FDA: Food and Drug Administration
KD: Dissociation Constant
CIA: Collagen-Induced Arthritis
SpA: Spondyloarthritis
PsA: Psoriatic Arthritis
Off-label: Use of a drug for an indication not approved by regulatory authorities
SKE: Skeletal muscle-restricted (engineered strain)
SpA: Spondyloarthritis
DAS28-ESR: 28-joint Disease Activity Score using the Erythrocyte Sedimentation Rate
VAS: Visual Analog Scale (for pain)
SSZ: Sulfasalazine
HAQ: Health Assessment Questionnaire
LPS: Lipopolysaccharide
